# Aging of a Poly(vinyl acetate)-Based White Glue and Its Durability in Contemporary Artworks

**DOI:** 10.3390/polym16121712

**Published:** 2024-06-15

**Authors:** Massimo Lazzari, Thais López-Morán

**Affiliations:** 1 Departamento de Química Física, Facultade de Química, Universidade de Santiago de Compostela, Avenida das Ciencias s/n, 15782 Santiago de Compostela, Spain; 2 Centro Singular de Investigación en Química Biolóxica e Materiais Moleculares (CiQUS), Universidade de Santiago de Compostela, 15782 Santiago de Compostela, Spain; 3 Centro Galego de Arte Contemporánea (CGAC), R. de Ramón del Valle-Inclán 2, 15703 Santiago de Compostela, Spain; cgac.conservacion@xunta.gal

**Keywords:** natural aging, oxidation, plasticizers, FTIR spectroscopy, surface enhanced Raman spectroscopy

## Abstract

While extensive research has focused on understanding the degradation mechanisms of Poly(vinyl acetate) (PVAC) paint under different environmental conditions, limited attention has been paid to the long-term stability of PVAC-based white glues, especially when used in artworks. This study investigates the accelerated degradation, under simulated photoaging, and isothermal treatment of a commercial PVAC-based white glue considered representative of this class of materials used in contemporary artworks to predict its durability and assess its behavior in art objects. Through accelerated aging experiments and comparison with natural aging observed in artworks, the study reveals the formation of chromophores and the release of plasticizers as key processes; in particular, the progressive darkening was considered an early indicator of degradation processes, before structural changes could be detected by FTIR or NMR spectroscopies. The plasticizer loss induces an increase in glass transition temperature, from 7 °C to temperatures higher than room temperature, affecting the adhesive’s cohesive strength and contributing to the detachment of materials in artworks. The findings underscore the importance of preventive conservation measures to mitigate degradation issues in PVAC-based artworks.

## 1. Introduction

Poly(vinyl acetate) (PVAC) is a rubbery polymer industrial which serves as the film-forming ingredient in water-based paints [1]. It is also used as an emulsion in the formulation of adhesives [2]. Stability issues of PVAC-based materials have been the subject of specific studies since the 1950s, on the degradative behavior under different environments, from thermal to thermo- and photo-oxidative artificial aging conditions [3]. At temperatures higher than 200 °C, PVAC easily releases acetic acid, leaving C=C double bonds on the main chain [4], a process that is also favored by UV irradiation [5]. Recent studies on oxidation under milder conditions, i.e., at lower temperatures or under simulated solar irradiation, showed good stability of PVAC, with chain scission as the principal degradation mechanism, with very limited deacetylation [6]. Specific works devoted to PVAC paints show how their complex chemical composition affects the degradation mechanism [3,7,8,9]. In particular, the presence of vinyl acetate copolymers and external plasticization, as usual in paint formulations, provoke lower stability, which may consist of a slight yellowing and a loss of plasticizer, inducing significant changes in mechanical properties [10]. More recently, the assessment of the conservation conditions of design objects made of extruded PVAC-based resin filaments and exposed to decades of natural aging showed the formation of aromatic and unsaturated fluorophores [11], related to the presence of plasticizer and the extensive deacetylation of the bulk polymer, respectively. Many studies also concerned the degradability of vinyl acetate-based adhesive used in works of art, for canvas lining, and other conservation practice applications such as consolidation of wall painting, wood, glass, paper, or metal [12,13,14,15,16] In general, the copolymeric nature of the main component in most commercial adhesives, e.g., copolymer of ethylene, (meth)acrylates, or other esters with vinyl acetate, does not modify the process of degradation, eventually accelerating its effects [12]. The main degradation route is still characterized by the loss of acetyl groups, while the less extended processes resulting from the competition between depolymerization and cross-linking have the effect of strongly affecting the adhesive solubility and removability.

On the other hand, information on the long-term stability of the so-called white glues, based on PVAC emulsions, is rather limited [17,18,19], especially when used either during the restoration practice or directly during the process of the creation of artworks. PVAC adhesives exposed up to 5 years to light aging with 40 W fluorescent lamps showed a higher degree and faster yellowing than acrylic adhesives used as a comparison, and also a higher surface acidity due to acetic acid release. Aging also induced a decrease in flexibility [18].

As a fact, the purpose of this work is to study the accelerated degradation of a commercial PVAC-based white glue used in contemporary artworks, intending to predict its durability and, more in general, give hints on their behavior in pieces of art in which this material may constitute the critical, more degradable, component. After identifying the polymeric components of two artworks showing extensive detachments and assessing their conservation conditions, with particular attention to components hurting the overall artwork aging, a reference PVAC-based glue was submitted to artificial aging. Polymer changes were periodically checked during the accelerated degradation, mainly by attenuated total reflectance–Fourier transform infrared (ATR-FTIR) spectroscopy, differential scanning calorimetry (DSC), visual observation, and gravimetric methods (either weight loss or insoluble determination). Finally, the most relevant markers of artificial degradation were compared with those detectable in the reference artworks.

## 2. Materials and Methods

### 2.1. Materials

The serial artwork *Palette* (from the portfolio *For Joseph Beuys*, edition of 90) was realized by Tony Cragg in 1986. It is made of a thick layer of small plastic granulates glued onto a wood support as a painter’s palette, with overall dimensions of 74 × 57 × 1.5 cm (Figure S1 in the Supporting Information). Analyses were carried out on 9 plastic granulates and 3 adhesive fragments, with a dimension of a few cubic millimeters, detached from the surface and gathered in the artwork case (Figure S2).

*Tierra, ladrillo y agua I, II, III e IV*, realized in 2001 by Dario Villalba, consists of bricks, gravel, soil, sand, and dry vegetal residues glued on 4 canvases, with dimensions 200 × 250 cm (Figure S3). Detached debris of different materials, including 4 whitish adhesive fragments (Figure S4), were used for identification analysis. The inorganic and vegetable debris were not the subject of this study and will not be discussed further.

The same commercial white glue used by Villalba, named *cola blanca uso general* (Rayt, Badalona, Spain), was bought in a local store and used as a model PVAC-based adhesive.

### 2.2. Accelerated Aging and Characterization Techniques

Films for aging treatments, with a thickness of 80–100 μm, were prepared by spreading the commercial emulsion onto glass slides for all the measurements except for transmission FTIR spectroscopy measurements where KBr disks were used as support. Samples were dried at room temperature for 48 h and then 24 h at 80 °C, until reaching a constant weight.

Accelerated photoaging treatment was performed in a high-speed exposure unit Suntest CPS+ (Heraeus), equipped with a xenon light source with constant irradiation at 765 W/m^2^. A glass filter with a cut-off at λ < 295 nm was used to exclude radiation more energetic than that of outdoor daylight exposure, in order to reproduce solar irradiation. The maximum temperature of the samples during irradiation was 44 °C black panel temperature, as maintained by a forced-air circulation system. Isothermal treatments were performed at constant temperatures in the range of 80–150 °C in a forced-air circulation oven.

FTIR spectra were collected using a Thermo Nicolet 6700 FTIR instrument equipped with a Smart Endurance device for ATR measurements and a liquid nitrogen-cooled mercury cadmium telluride (MCT) detector (spectral range 4000–670 cm^−1^) at 4 cm^−1^ resolution for 128 scans. Data were processed with Omnic 8.1 by Thermo Nicolet. Samples from artworks were analyzed as received. Color measurements were performed with a portable Konica Minolta CM 700d spectrophotometer (Konica Minolta, INC., Tokyo, Japan). Color space was the CIELAB with D65 standard illuminant and 10° observer, ø 6 mm illumination area. The International Commission on Illumination (CIE) developed this L*a*b* color model in 1976 to measure objective color and calculate color differences. DSC thermograms were obtained with a Q200 (TA Instruments, New Castle, DE, United States) calorimeter equipped with a refrigerated cooling system in the temperature range from −70 to 100 °C, using 10–15 mg samples with a scanning rate of 20 °C min^−1^, under a 50 mL min^−1^ nitrogen flow. Weight changes in supported films and insoluble fractions were determined gravimetrically with an analytical balance and Gr-200 with a 0.0001 g repeatability and ±0.0002 g linearity. Surface-enhanced Raman spectroscopy (SERS) spectra were recorded with a Renishaw InVia Flex Raman spectrometer (Wotton-under-Edge, UK) equipped with a continuous wave laser with emission at 514 nm with gratings of 1800 lines mm^−1^. Measurements were performed with a long 0.50 NA NPlan long working distance objective Leica 566036 (Leica Microsystems Gmbh, Wetzlar, Germany) operating with a 65 µm slit opening, with a 10 s accumulation and 1% of the nominal power, corresponding to 0.09 mW. The spectrometer slit opening was 65 µm, using the detector Renishaw CCD 576 × 400 pix. Sampling from aged surfaces was carried out by 0.5 × 0.5 cm silicone strip samplers fabricated by casting and thermal curing of a liquid prepolymer (Silgard 184, Dow Corning, Midland, MI, United States) under vacuum at 80 °C for 2 h. Physisorbed molecules were then dissolved in around 10 µL of chloroform and transferred onto SERS-active Al-coated 3D nanostructures. More details on the sampling procedure and the fabrication of the SERS substrates were reported elsewhere [20,21].

## 3. Results

### 3.1. Materials Characterization

**Figure 1:**
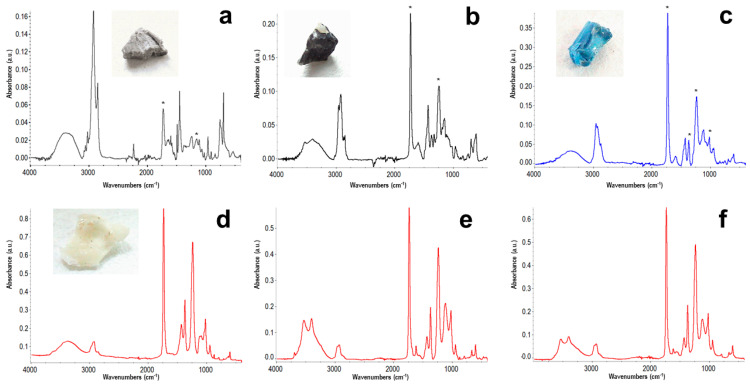
ATR-FTIR spectra of fragments of ABS (**a**), PP (**b**), and PE (**c**) (marks indicate main PVAC peaks), and an adhesive fragment (**d**) from *Palette* by T. Cragg, compared with that of adhesive debris from Villalba’s artwork (**e**), and the transmission FTIR spectrum of dried commercial glue (**f**).

A total of 9 plastic granulates and 3 adhesive fragments released from the artwork *Palette* were analyzed by ATR-FTIR spectroscopy to identify the polymeric components and enlighten whether aging may justify their detachment from the support. As typical in Cragg’s work [22], the granulates consisted of common industrial polymers, such as polyethylene (PE), polypropylene (PP), polystyrene, and acrylonitrile-butadiene-styrene copolymer (ABS) (examples in Figure S2). They did not show any relevant sign of degradation but were always coated with a thin layer of adhesive on the surface. Common features are marked in the examples of ATR-FTIR spectra shown in Figure 1a–c. In addition, the spectra of fragments of the adhesive (example in Figure 1d) allowed the identification of PVAC as its main component. The main peaks at 3000–2800 cm^−1^ (CH_2_ and CH_3_ stretching), 1735 cm^−1^ (C=O stretching), 1434 and 1372 cm^−1^ (CH_2_ and CH_3_ bending), and 1023 cm^−1^ (C-O stretching) are due to PVAC. The broad –OH stretching absorption in the 3600–3100 cm^−1^ is associated with poly(vinyl alcohol) [23], a typical secondary component of PVAC-based white glues.

Also, in the case of Villalba’s artwork, the ATR-FTIR spectrum of the whitish debris (Figure 1e) shows the series of peaks detailed for Cragg’s adhesive, with only some difference in the relative intensity of some minor peaks, which allowed to identify the adhesive used for its assembly as a PVAC-based glue. In particular, with respect to Cragg’s glue residue, the spectrum in Figure 1e shows a more complex OH absorption band and other minor peaks, e.g., at approximately 1620 cm^−1^, indicating additional glue components. In addition, the higher relative intensity of the peak at around 1100 cm^−1^ with respect to the PVAC C-O stretching peak at 1023 cm^−1^ in Figure 1e in comparison with Figure 1d can be related to a different amount of plasticizers [23].

PVAC emulsions used as adhesives essentially consist of a PVAC homopolymer produced in the presence of a polymeric protective colloid, usually low molecular weight PVOH, and other additives, such as plasticizers and coalescence agents [24,25]. Despite compositional differences within this class of adhesives, as recently studied by De Sá et al. [23], long-term aging is expected to follow common features. In this work, we considered the behavior of the commercial white glue stated by Villalba during a private conversation, as representative of the entire class.

The technical sheet of the Rayt commercial white glue only indicates the solid fraction (approximately 50 wt.%), PVAC as the main component, PVOH as a minor component, gypsum (CaSO_4_·2H_2_O), and other unidentified additives. Elemental analysis, which detected a high amount of sulfur, and TGA allowed to confirm the presence of gypsum and its quantification (19 wt.%), while GC/MS unveiled the presence of dimethyl succinate and dibutyl phthalate (DBP).

### 3.2. Accelerated and Natural Aging

To predict the long-term degradative behavior of the critical adhesive material in the artworks and better understand its loss of cohesive strength, dry films of the commercial glue were exposed to simulated aging conditions [26]. As a common practice in polymer science, all the processes occurring during natural aging may be artificially accelerated through either (a) irradiation in a photoaging device equipped with a solar lamp or (b) isothermal treatment at a temperature higher than the environmental one, not triggering unexpected reactions in both cases. Under such limitations, accelerated aging treatments are only expected to induce the same chemical and physical changes, including oxidation and additives’ migration, at a higher rate than those occurring under museum conditions [22].

Reference samples in the form of films were prepared by water evaporation onto a solid substrate, either glass or quartz, and dried until constant weight. Differently from the case of pure PVAC homopolymer, our dried films showed a very limited solubility in common organic solvents such as chloroform and acetone, thus suggesting an emulsion formulation also containing small amounts of a cross-linker. In addition, by comparison with the adhesive fragments sampled from the artworks (Figure 1d,e), the transmission FTIR spectrum of the dried reference adhesive (Figure 1f) shows the presence of two small peaks at approximately 1580 and 1540 cm^−1^ due to the plasticizer, i.e., DBP identified by GC/MS.

Films submitted to photoaging showed negligible weight loss for treatment up to 1500 h (<1%), whereas the surface turned from transparent to white already after 500 h due to the so-called blooming. Both phenomena may be related to the partial migration of the plasticizer, i.e., DBP, to the surface, and then partially evaporating. As a further confirmation of the fact that a complete release of DBP from a PVAC matrix has been observed only after 2000 h aging in the same type of photodegradation device [6], transmission FTIR spectra of 1500 h aged films only showed minor modification in the range 1600–1500 cm^−1^, which is difficult to be quantified.

Due to the high stability of PVAC-based materials [6,10,12], we also carried out simulated aging at isothermal conditions, at temperatures high enough to accelerate the degradation processes at a higher rate than photoaging in solar lamp devices. In addition, previous studies by Allen and co-workers did not show changes at 120 °C [27]. On the other hand, our isothermal treatment essays at higher temperatures entailed the same changes observed at 130 °C as detailed below, but in a much shorter aging time.

**Figure 2:**
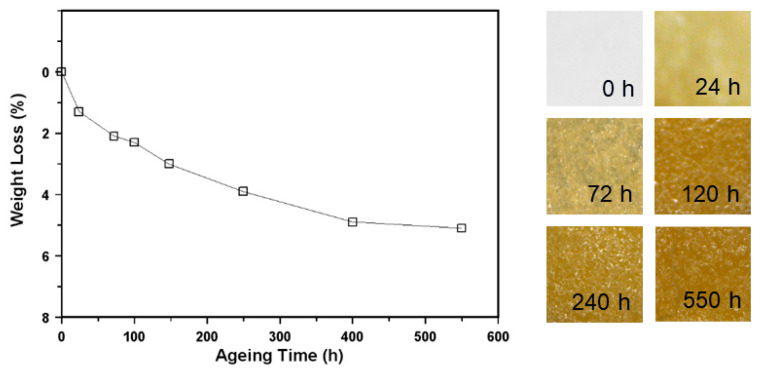
Weight loss of dried commercial glue films exposed to isothermal aging at 130 °C as a function of treatment time, and photographs showing the corresponding color changes.

**Figure 3:**
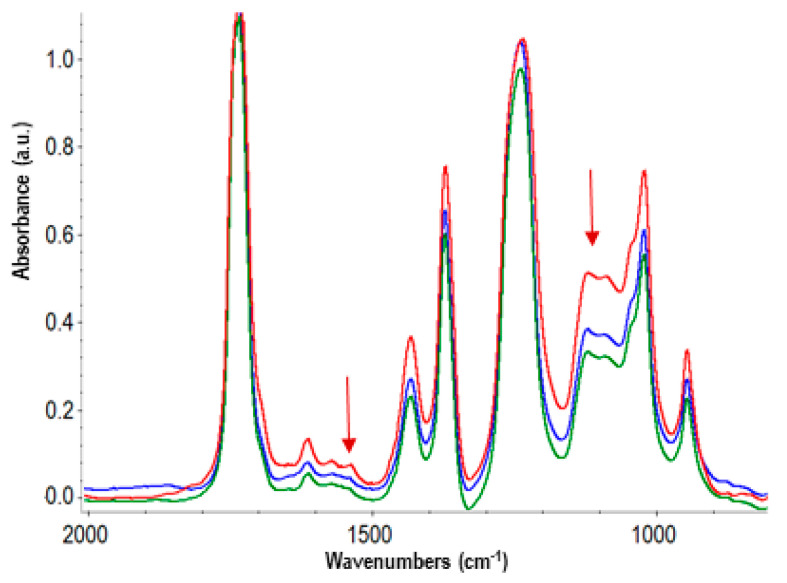
Transmission FTIR spectra in the 2000–800 cm^−1^ of commercial glue films before (red line) and after 120 h (blue) and 550 h (green) isothermal aging at 130 °C.

The weight loss during the isothermal treatment at 130 °C was more marked than during the photoaging and increased over time, up to a final 5% loss after 550 h (Figure 2). Even if, in principle, the partial decomposition of gypsum with crystallization water release cannot be excluded, achieving the same plateau value also at higher constant temperature treatments revealed the release of some other molecules. The corresponding chemical changes were monitored by FTIR spectroscopy, showing small spectral modifications very similar for the treatments at 130 °C and higher temperatures (selected spectra are shown in Figure 3). They essentially consist of a decrease in the relative intensity of some peaks or shoulders, which may be related to the volatilization of the plasticizer. In particular, the decrease in the 1580 and 1542 cm^−1^ peaks until their virtual disappearance, together with the decrease in shoulders/secondary peaks at approximately 1460, 1120, and 1075 cm^−1^. No new peaks were formed during the isothermal treatment for up to 550 h, confirming the good oxidative stability of PVAC [28], nor were there signs of the well-known process of deacetylation that would lead to the formation of C=C double bonds [10]. Moreover, even the use of a technique more sensitive than FTIR spectroscopy to detect the formation of new groups, such as nuclear magnetic resonance, forcedly at the solid state, did not offer further information.

At the same time, the visual inspection of the aged films showed an extensive color change since the beginning of the isothermal treatment (Figure 2), quantified using a spectrophotometer in the CIELAB color space (Table S1). *ΔE* reaches a high value from 24 h up to approximately 45 at 550 h. A more detailed analysis of the evolution of L*, a*, and b* coordinates indicates that the most relevant variations are those of ΔL* and Δb*, indicating a decrease in luminosity and a progressive yellowing, respectively. The formation of chromophores is considered an early indicator of degradation processes [22]. Although not confirmed, the progressive darkening is due to the formation of increasingly longer sequences of C=C double bonds over time, although in an amount not detectable by FTIR spectroscopy.

**Figure 4:**
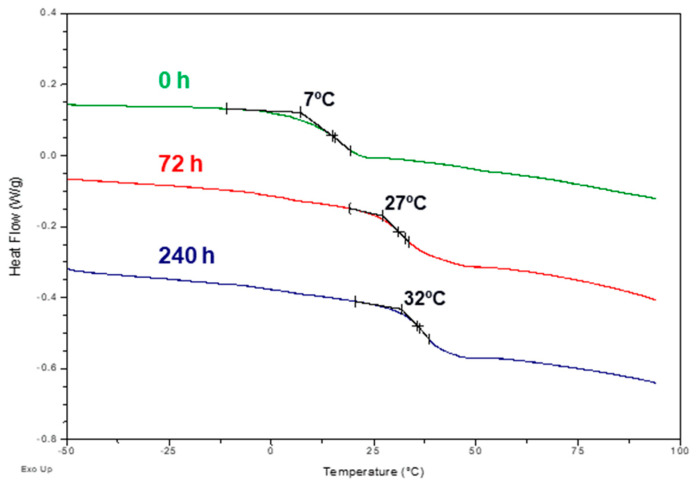
Selected DSC thermograms.

The evolution of the film’s aging may additionally be followed through an indirect evaluation of the changes influencing molecular motion by DSC. The temperature of glass transition, T_g_, of PVAC homopolymer is in the range of 35–40 °C, depending on the molecular weight [29], but it is expected to be much lower in dried white glue due to the presence of PVOH and other low molecular weight additives, especially plasticizers. During the isothermal treatment at 130 °C, the T_g_ increases from an initial value of 7 °C to 35 °C after 550 h aging (Table 1, with examples of thermograms in Figure 4). This behavior may directly be related to the release of the plasticizer, thus confirming FTIR spectroscopy highlights. Films submitted to photoaging only showed negligible T_g_ changes.

**Figure 5:**
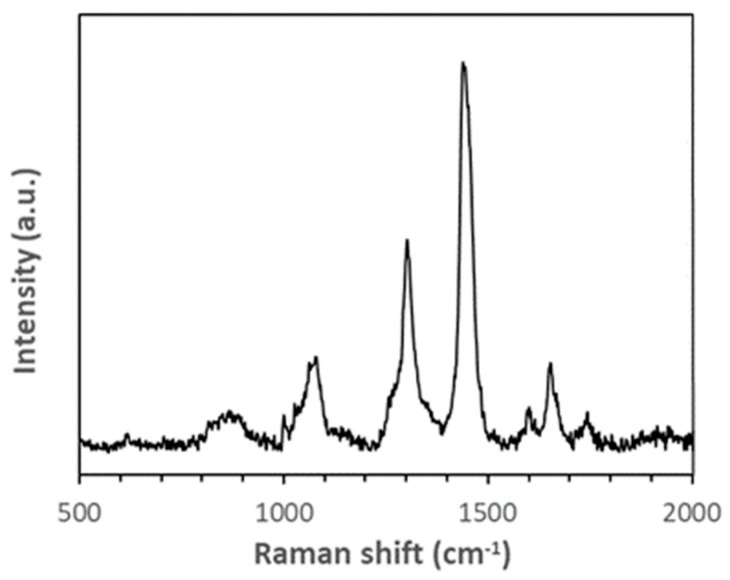
SERS spectrum in the 500–2000 cm^−1^ range of surface molecules from dried commercial glue films exposed to isothermal aging at 130 °C for 24 h.

A direct indication of the release of DBP was obtained through a surface analysis strategy based on SERS measurements, which was developed to identify degradation markers with low molecular weight from polymer surfaces [21] and further optimized to detect small molecules of dyes and pigments from prints. Films were sampled using silicone strip samplers pressed onto the exposed film surface for 30 s. The low molecular weight molecules eventually physisorbed by the sampler were then dissolved in chloroform and transferred onto Al-coated nanostructured SERS-active substrates. Further details on the substrates, with an enhancement factor of 10^9^ at the excitation wavelength of election, i.e., 514 nm, their fabrication, and use are reported elsewhere [20]. Dried films before aging did not reveal the presence of any molecules on their surface, whereas SERS spectra of surface molecules from aged films after at least 100 h photoaging or 24 h isothermal treatment at 130 °C showed spectra like that shown in Figure 5. Even if an unambiguous identification is impossible, characteristic peaks at approximately 1745, 1602, and 1060 cm^−1^ are compatible with phthalates [30]. This hypothesis agrees with the features highlighted by FTIR spectroscopy and DSC, justified with a plasticizer loss without showing any structural changes associated with PVAC degradation.

Validation of the results from monitoring the accelerated aging of the commercial white glue was carried out via direct comparison with its natural aging under milder museum conditions for more than 20 or 30 years in the two selected artworks. As it was already clear that FTIR spectra of the adhesive used in artworks did not contain appreciable amounts of plasticizers, i.e., no peaks in 1600–1500 cm^−1^ in the spectra in Figure 1d,e, such adhesive fragments were also submitted to DSC measurements to compare their molecular mobility with that of the “as applied” glue, immediately after drying. In both cases, the thermograms showed T_g_ higher than the initial, and precisely 16 °C and 30 °C for the Villalba and Cragg’s adhesive, respectively (example in Figure S5).

PVAC tends to creep under a sustained load [31], and adding a plasticizer in its formulation has, among others, the effect of lowering hardness and strength and increasing creep [2]. Thus, increasing adhesion is a fundamental characteristic to guarantee the stability of the works of art, especially in the case of Villalba’s work, based on the assembly of heavier objects. The observed loss of plasticizer, consequent T_g_ and hardness increase, and partial loss of creep strength during aging fit perfectly with the detachment of debris with different weights from the artworks. Concerning their visual appearance, it is also worth noting that artwork fragments also showed extensive surface whitening, as seen during the aging simulation. In addition, contrary to what was observed during isothermal treatments, they did not show any yellowing. This behavior may be due to the fact that longer times of natural aging are necessary to accumulate a concentration of chromophores, tentatively sequences of C=C double bonds, high enough to be visible.

## 4. Conclusions

Based on the abovementioned experimental evidence, the commercial PVAC-based glue, selected as a behavioral model of the white glues used by Villaverde and Cragg, showed excellent stability during accelerated aging. The PVAC component did not show any structural modifications detectable by FTIR or NMR spectroscopies except for the formation of chromophore structures that are not otherwise detectable, tentatively containing conjugated C=C double bonds. The only detectable molecular changes in the dried films were associated with the volatilization of a low molecular weight component, the DBP, which has the effect of inducing an increase in the T_g_ from 7 °C to temperatures higher than ambient conditions. Concerning the natural aging of glues, the most relevant sign consists of the hardness increase in the dried glue due to plasticizer loss, which induces a loss of creep, especially under the higher load in the artwork of Villalba than in the older Cragg’s assembly. Finally, it may be predicted that both artworks, and many others containing white glues, will further suffer from these conservation problems in the coming years, unless urgent preventive conservation measures are applied.

## Figures and Tables

**Table 1:** Evolution of the T_g_ of dried commercial glue films exposed to isothermal aging at 130 °C.

Time (h)	T_g_ (°C)
0	7
24	19
72	27
120	30
240	32
550	35

## Data Availability

Data are contained within the article and Supplementary Materials.

## Supplementary Materials

The following supporting information can be downloaded at: https://www.mdpi.com/article/10.3390/polym16121712/s1. Figure S1: Photograph of *Palette* (from the portfolio *For Joseph Beuys*) by Tony Cragg; Figure S2: Detail of *Palette* and photographs of detached debris; Figure S3: Photograph of *Tierra, ladrillo y agua I, II, III e IV* by Dario Villalba; Figure S4: Details of the Villalba’s artwork, also showing glue accumulation and detachments; Figure S5: DSC thermogram of Villalba’s white glue fragment; Table S1: Evolution of the CIELAB coordinates of dried commercial glue films exposed to isothermal aging at 130 °C.
