# Supplementary material for: Aging of a Poly(vinyl acetate)-Based White Glue and Its Durability in Contemporary Artworks
Source: Polymers (Basel). 2024 Jun 15;16(12):1712. doi: 10.3390/polym16121712 (PMC11207482; doi:10.3390/polym16121712)
Supplement: Supplementary file 1 [file polymers-16-01712-s001.zip › polymers-2916911-supplementary.pdf]

Aging of a poly(vinyl acetate)-based white glue and its durability in contemporary artworks

Massimo Lazzari [1,2,*], **Thais López Morán** [3]

[1] Departamento de Química Física, Facultade de Química, Universidade de Santiago de Compostela, Avenida das Ciencias s/n, 15782 Santiago de Compostela, Spain;
[2] Centro Singular de Investigación en Química Biolóxica e Materiais Moleculares (CiQUS), Universidade de Santiago de Compostela, 15782 Santiago de Compostela, Spain;
[3] Centro Galego de Arte Contemporánea (CGAC), R. de Ramón del Valle-Inclán 2, 15703 Santiago de Compostela, Spain; cgac.conservacion@xunta.gal

* Correspondence: massimo.lazzari@usc.es

Supplementary Materials: Figure S1: Photograph of *Palette* (from the portfolio *For Joseph Beuys*) by Tony Cragg; Figure S2: Detail of *Palette* and photographs of detached debris; Figure S3: Photograph of *Tierra, ladrillo y agua I, II, III e IV* by Dario Villalba; Figure S4: Details of the Villalba's artwork, also showing glue accumulation and detachments; Figure S5: DSC thermogram of Villalba's white glue fragment; Table S1: Evolution of the CIELAB coordinates of dried commercial glue films exposed to isothermal aging at 130ºC.

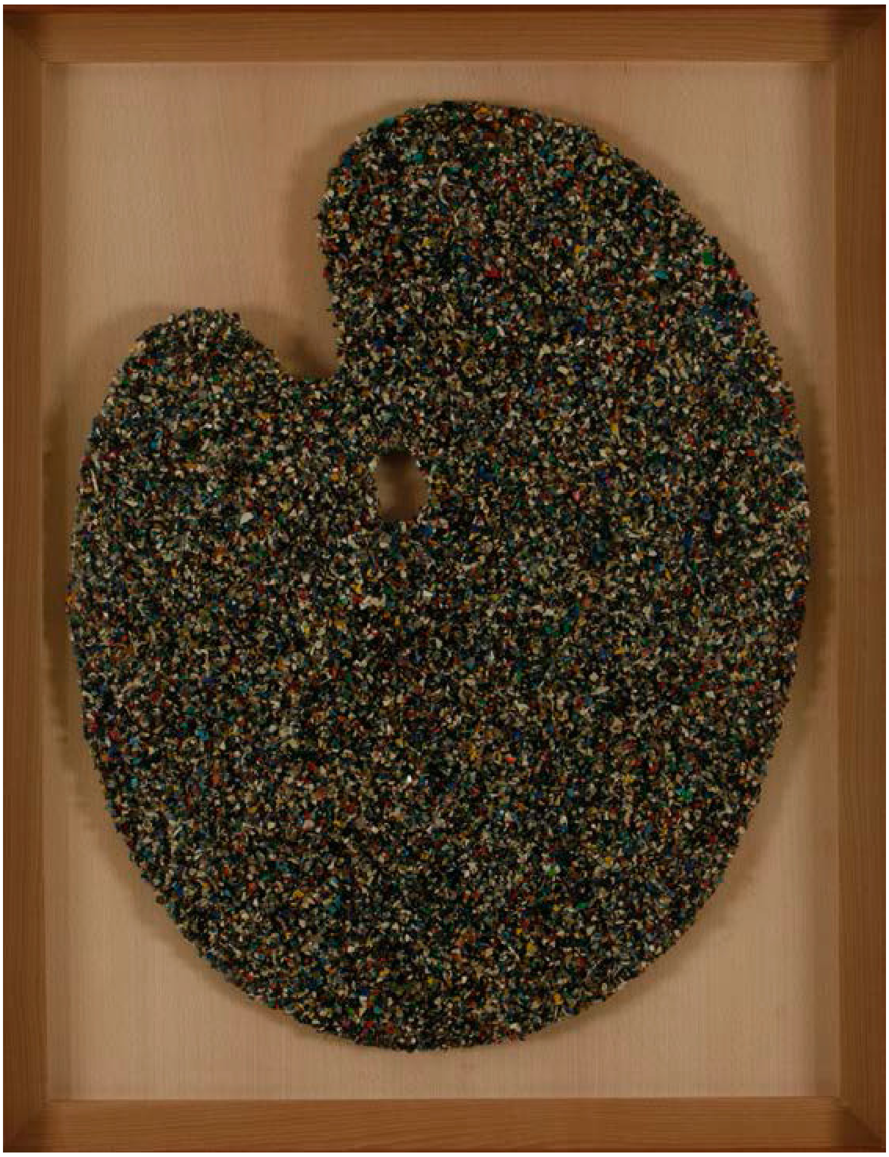

Figure S1. Photograph of *Palette* (from the portfolio *For Joseph Beuys*) by Tony Cragg (artwork of the permanent collection of the Centro Galego de Arte Contemporánea, CGAC).

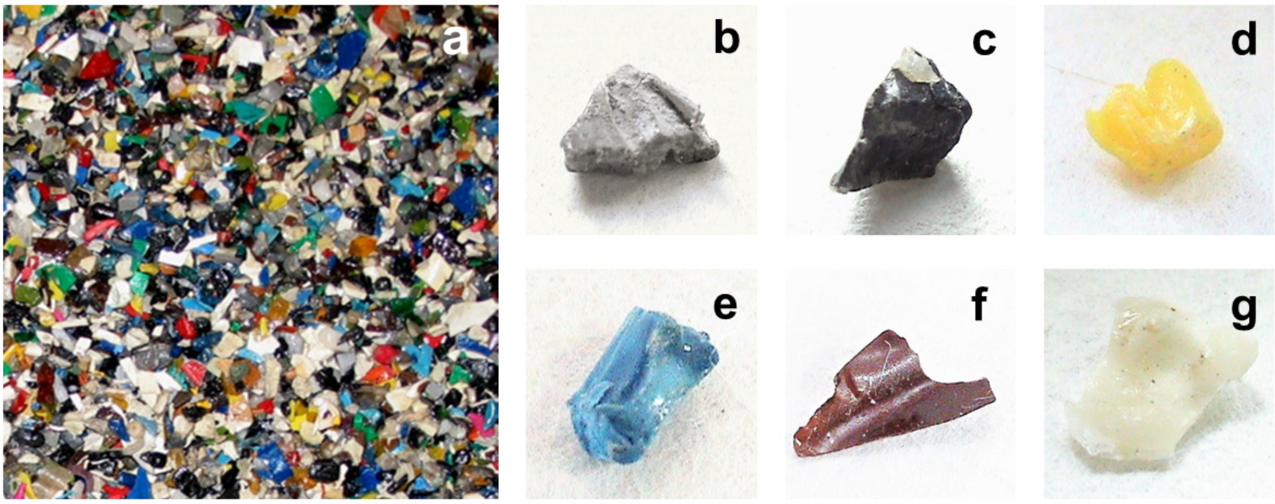

Figure S2. Detail of *Palette* (a) and photographs of detached debris (b-g).

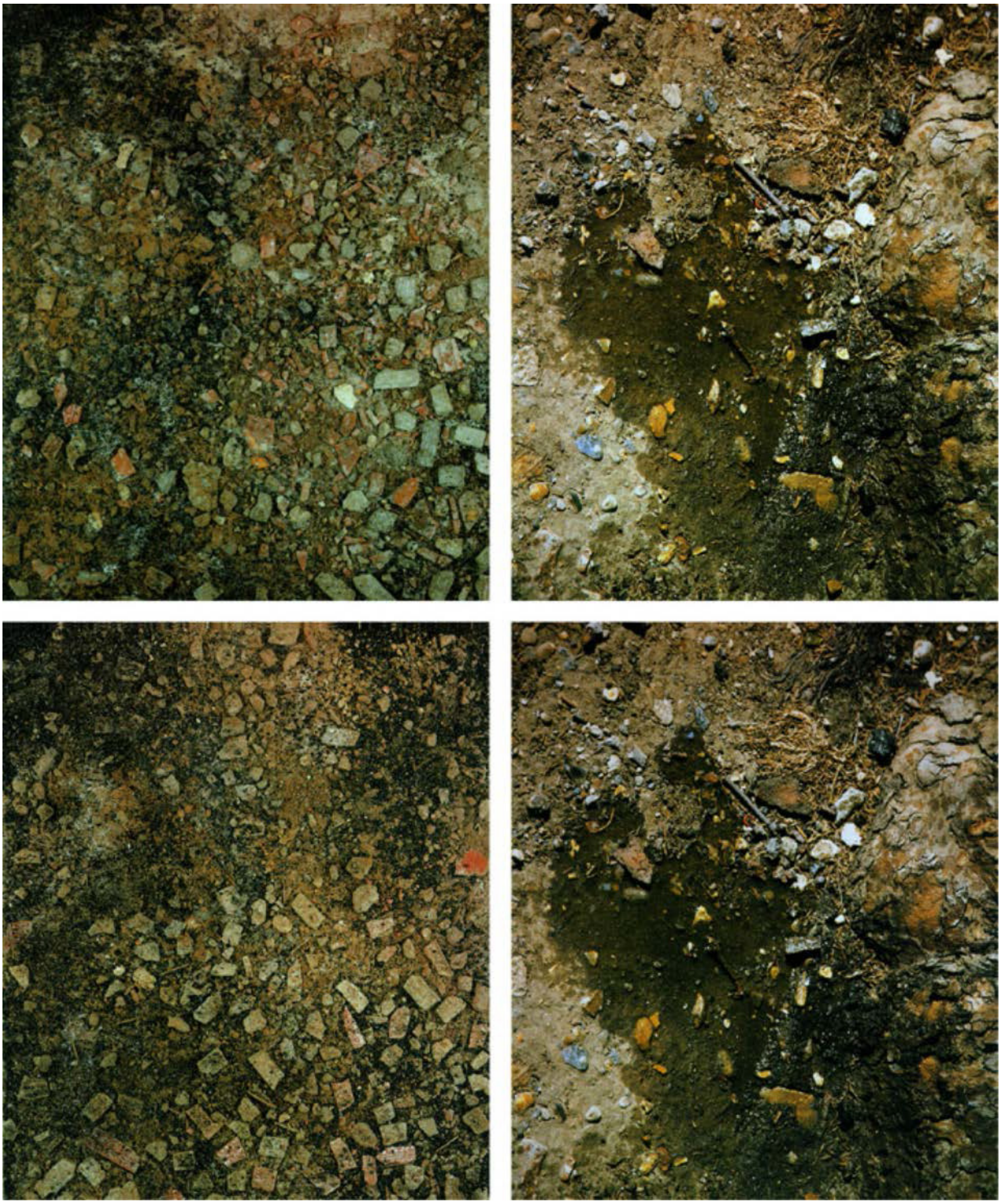

Figure S3. Photograph of *Tierra, ladrillo y agua I, II, III e IV* by Dario Villalba (artwork by the Centro Galego de Arte Contemporánea, CGAC).

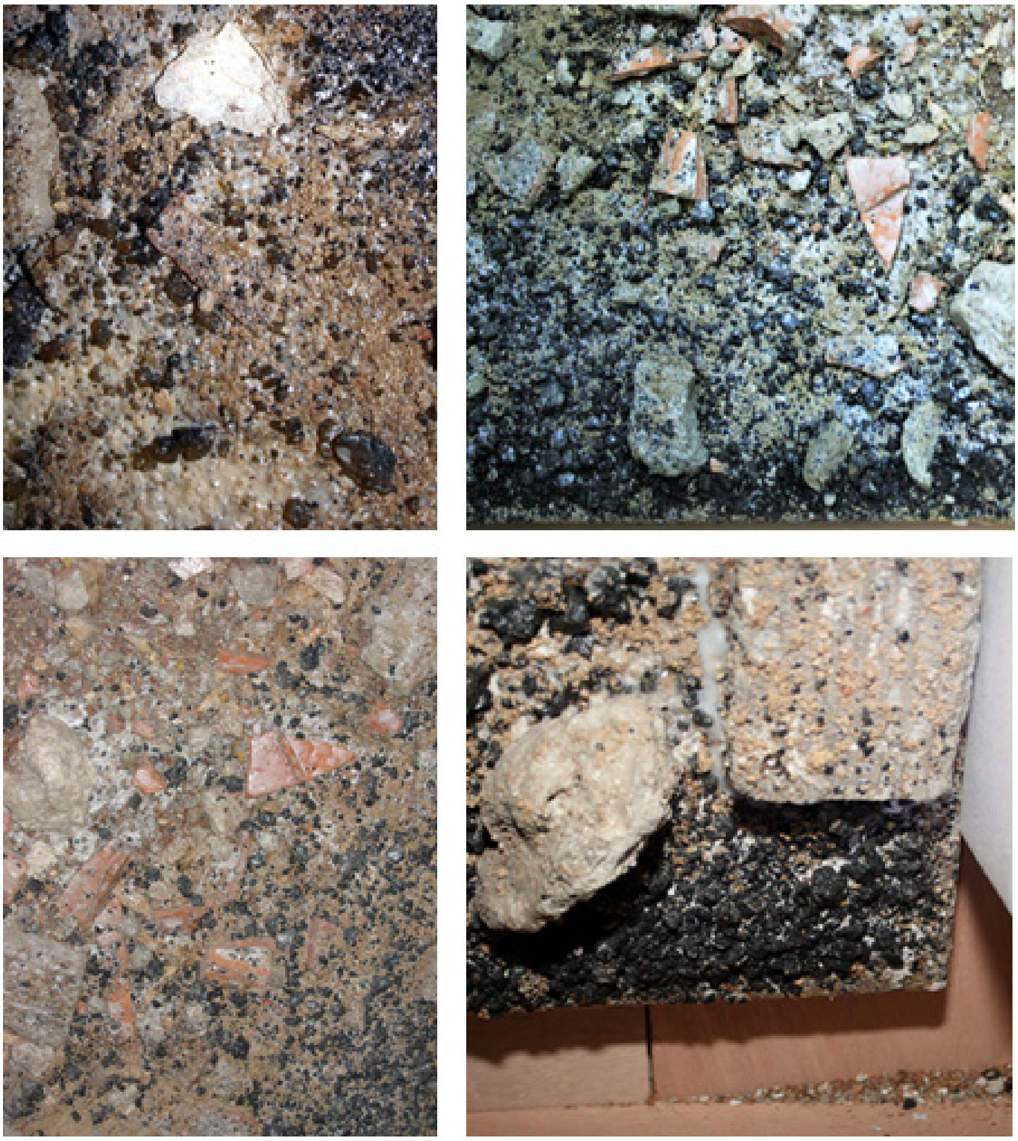

Figure S4. Details of the Villalba's artwork, also showing glue accumulation and detachments.

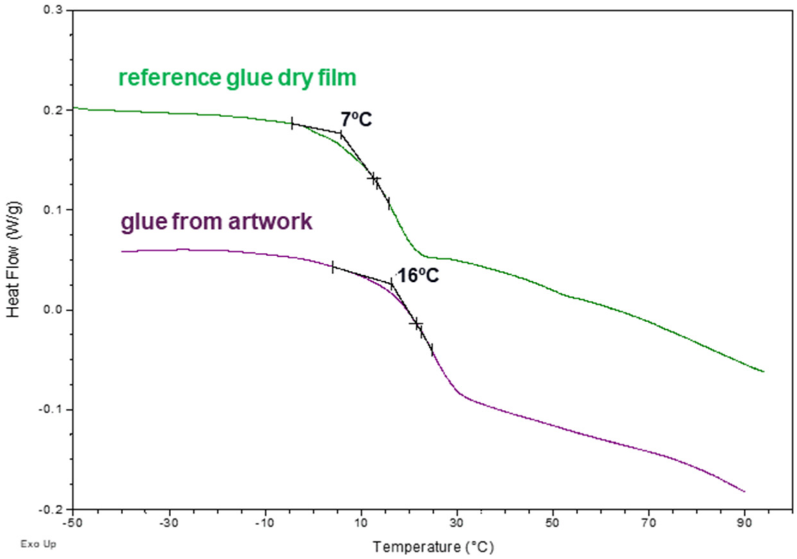

Figure S5. DSC thermogram of Villalba's white glue fragment.

Table S1. Evolution of the CIELAB coordinates of dried commercial glue films exposed to isothermal aging at 130ºC.

Time (h)	ΔL*	Δa*	Δb*	ΔE
24	-5.52	-9.98	15.43	19.19
72	-10.89	-9.54	18.82	23.74
120	-21.39	-9.89	25.00	34.36
240	-25.61	-8.48	29.84	40.23
550	-29.58	-8.87	32.41	44.77
